# A Bacterial Toxin that Causes DNA Damage to Modulate Cellular Responses

**DOI:** 10.1100/tsw.2001.27

**Published:** 2001-05-01

**Authors:** Maria Lara-Tejero

**Affiliations:** Section of Microbial Pathogenesis, Boyer Center for Molecular Medicine, Yale School of Medicine, New Haven, CT 06536, USA

**Keywords:** bacterial Toxins, cell cycle arrest, bacterial pathogenesis

## Abstract

Campylobacter jejuni constitutes the leading cause of bacterial diarrhea in the U.S. and all around the world [1]. This common bacterium produces a toxin known as cytolethal distending toxin (CDT) [2] which causes intoxicated cells to enlarge and to stop dividing with a double DNA content characteristic of G2/M arrest [3]. The effect of the toxin on the cell is so striking that it captivated scientists for a long time. However, its mechanism of action had remained elusive.

A recent paper published in the journal *Science* [4] provided important clues about the mechanism of action of CDT. It turns out that the active subunit of the CDT toxin is a nuclease. CDT- intoxicated cells possess double DNA content and accumulate the inactive hyperphosphorylated form of Cdc2, the cyclin-dependent kinase (CDK) responsible for triggering entry into mitosis [3]. Therefore, these cells are arrested prior at the G2/M boundary. Expression of three genes, *cdtA, cdtB,* and *cdtC* was known to be required for CDT activity [5]. However, the individual contribution of each of the gene products to the toxic phenotype or the mechanism of action of the CDT holotoxin was unknown. Analogous to other bacterial toxins, the hypothesis was that CDT would have at least one catalytically active component, and at least one component required for the delivery of the active subunit into the target cell. For many toxins, delivery or direct expression of the active subunit inside the target cell bypasses the need for the delivery subunit to induce toxicity. With this hypothesis in mind, the individual Cdt proteins were expressed in eukaryotic cells as an attempt to obtain information about the putative active subunit of the CDT toxin. Expression of CdtB led to catastrophic changes in the chromatin of the transfected cell whereas expression of CdtA or CdtC did not result in any overt detrimental effect to the cell. These results suggested that the CdtB protein could be the active subunit of the CDT toxin. Examination of the CdtB sequence using amino-acid threading to proteins whose structures are presently known revealed a very significant structural homology to DNase I. This similarity, in conjunction with the transient expression phenotype, led to the hypothesis that maybe CdtB was exerting its toxic effect as a nuclease. Consistent with this hypothesis, point mutation in residues that were predicted to be essential for a putative DNase I-like activity of CdtB effectively abolished its ability to induce any chromatin changes when transiently expressed in eukaryotic cells. Further experiments showed that the ability of CdtB to damage the cell chromatin strictly correlated with CDT toxicity. Bacterial lysates expressing a CDT holotoxin containing a CdtB mutant component were not able to induce the distention and cell cycle arrest characteristic of CDT intoxication. This was consistent with the hypothesis that the putative nuclease activity of CdtB was essential for CDT activity. Additional experiments showed that microinjection of very small amounts of purified CdtB could recapitulate in its entirety the effects of the CDT holotoxin including cytoplasm distention and cell cycle

Contact info: Phone: (203) 737–2407; Fax: (203) 737–2630
E-mail: maria.lara-tejero@yale.edu
© 2001 with author.

arrest. This set of experiments demonstrated that CdtB is the active subunit of the CDT holotoxin. The following mechanism of action for the CDT toxin was proposed: upon delivery into the target cell, most likely aided by CdtA and/ or CdtC, CdtB gains access to the nucleus of the cell where it inflicts limited damage to the DNA that is insufficient to kill the cell, but sufficient to trigger the DNA damage checkpoint responsible for the observed cell cycle arrest.

It is becoming increasingly clear that a number of bacterial pathogens have evolved sophisticated strategies to modulate cellular functions. These strategies are the result of co-evolutionary forces aimed at securing the survival of both the host and the pathogen. The cytolethal distending toxin is a remarkable example of such a strategy. By inflicting limited damage to the cell, CDT triggers a response, cell cycle arrest which presumably benefits the pathogen.

Finally, the presence in such a common intestinal pathogen of a toxin able to cause DNA damage raises the intriguing possibility that CDT could cause genomic instability in intestinal cells thereby contributing to the development of intestinal cancer. Exciting studies undoubtedly to come will address some of the intriguing questions raised by these studies.
